# Restorative and Esthetic Dentistry—A Special Issue of the *Dentistry* *Journal*

**DOI:** 10.3390/dj6010005

**Published:** 2018-02-11

**Authors:** Min-Huey Chen

**Affiliations:** 1 Graduate Institute of Clinical Dentistry, School of Dentistry, National Taiwan University, Taipei 10617, Taiwan; minhueychen@ntu.edu.tw; Tel.: +886-2-23123456 (ext. 67701); 2 Department of Dentistry, National Taiwan University Hospital, Taipei 10617, Taiwan

Due to the development of dental materials and the esthetic requirements of patients, conservative restoration and esthetic dentistry is becoming more and more important. It is therefore timely to create a platform for all researchers and clinicians to share their experiences and studies in the field of “Restorative and Esthetic Dentistry”. To promote the development of this field and provide readers with updates in this area, *Dentistry Journal* has set up this Special Issue on “Restorative and Esthetic Dentistry”. All studies about restorative and esthetic dentistry are welcome, including clinical case reports and studies on dental materials. Topics welcome in this issue include: veneer, tooth colored inlay/onlay, bleaching, full ceramic crown, oral rehabilitation of vertical dimension loss, direct and indirect composite resin restorations, dentin bonding agents, resin cement, zirconia implant, vital pulp therapy, Biodentine, Mineral trioxide aggregate (MTA), resin adhesive bridge, Maryland bridge, gingival esthetics, digital impression, laser cavity preparation, fiber post, and conservative restoration.

## Keywords

- veneer
- tooth colored inlay/onlay
- ceramic inlay/onlay
- composite resin inlay/onlay
- bleaching
- full ceramic crown
- oral rehabilitation of vertical dimension loss
- composite resin restoration
- fiber reinforced composite resin
- direct and indirect composite resin restorations
- dentin bonding agents
- dental adhesives
- resin cement
- zirconia implant
- vital pulp therapy
- Biodentine
- Mineral trioxide aggregate (MTA)
- dental wear
- dentin hypersensitivity
- resin adhesive bridge
- Maryland bridge
- gingival esthetics
- digital impression
- laser cavity preparation
- fiber post
- conservative restoration

